# Transition to Motherhood: A Study on the Association between Somatic Symptoms during Pregnancy and Post-Partum Anxiety and Depression Symptoms

**DOI:** 10.3390/ijerph191912861

**Published:** 2022-10-07

**Authors:** Renata Tambelli, Giulia Ballarotto, Carmen Trumello, Alessandra Babore

**Affiliations:** 1 Department of Dynamic and Clinical Psychology, and Health Studies, Sapienza University of Rome, 00185 Rome, Italy; 2 Department of Psychological, Health and Territorial Sciences, D’Annunzio University of Chieti-Pescara, 66100 Chieti, Italy

**Keywords:** motherhood, post-partum, depression, anxiety, fear of childbirth, nausea, vomiting, sleepiness

## Abstract

Several authors found that somatic symptoms during pregnancy such as nausea, vomiting, and levels of sleep, and fear of childbirth were associated with women’s post-partum psychopathological difficulties. The present study aimed to verify whether fear of childbirth can mediate the relationship between some somatic symptoms experienced during pregnancy (i.e., nausea, vomiting, and daily sleep duration) and the post-partum depressive and anxious symptoms. *N* = 258 mothers of children between 3 and 6 months of age filled out self-report questionnaires assessing somatic symptoms during pregnancy, fear of childbirth, and anxious and depressive symptoms during post-partum. Results showed that levels of vomiting during pregnancy (but not nausea and daily sleep duration) was associated with post-partum depression and anxiety. Furthermore, findings showed that fear of childbirth partially mediated the relationships between the levels of vomiting during pregnancy and post-partum state anxiety and depression. These results can have several clinical implications, allowing to implement preventive programs for post-partum depression, considering vomiting and fear of childbirth as important risk factors.

## 1. Introduction

The Stern’s notion of the motherhood constellation [1] symbolizes a “new psychic organization”, a process by which the woman acquires her own maternal role, that begins during maternal pregnancy, a period in which the mother thinks about and modifies the representations of herself as a woman and as a mother. At the same time, the mother experiences several bodily changes and she can present some physical symptoms, such as excessive sleepiness, nausea, and vomiting. Several authors have hypothesized that some somatic symptoms occurring during pregnancy might represent psychosomatic symptoms of an intrapsychic difficulty in acquiring the maternal role and be associated with a following psychopathological risk [2,3,4]. 

Nausea and vomiting during pregnancy (NVP) are symptoms that affect 50–90% of pregnant women [5,6]. These symptoms occur at the beginning of the first trimester, with a peak at around 9 gestational weeks and typically a decrease around at 20 gestational weeks [6], but in 5–22% of affected women the symptoms persist throughout pregnancy [7]. Increasing studies are focusing on the prolonged form of NVP extending in the second and third trimester of pregnancy [3,8,9,10]. Despite no clear etiology of NVP, it is widely accepted that gestational vomiting results from various metabolic and endocrine factors [11], such as human chorionic gonadotropin (hCG). From a bio-psycho-social perspective, several studies have investigated the role of psychological factors in association with biological mechanisms that cause symptoms such as nausea, vomiting, and sleepiness [8,9,10,12,13].

Recently, research underlined that women with prolonged NVP showed an increased risk of depressive symptoms after giving birth [10,14], and anxiety [3,8]. However, it is important to underline that in these studies nausea and vomiting are always considered together as a single symptomatic manifestation.

Furthermore, some somatic symptoms (such as insomnia, daily lassitude, and daily sleepiness) have been identified as valid indicators of depression during pregnancy [12,13], while sleep quality during the first trimester and third trimester were associated to post-partum depression [15,16]. 

If on the one hand several authors found somatic symptoms during pregnancy to be associated with women’s post-partum psychopathological difficulties [4,17,18], on the other hand, several authors have identified fear of childbirth to be an important linking factor between the concrete aspects of pregnancy and the psychological difficulties the woman may face [19,20,21]. 

Fear of childbirth is related to fear of the pain that women are going to feel during delivery [19,20], but also to fear of giving birth to a baby with congenital defects [22] or about problems during delivery that can compromise their life or health [23]. The prevalence of fear of childbirth in Europe varies from 1.9% to 14%, based on the country [24]. It is common for pregnant women to experience fears relating childbirth [25], and some level of fear may be considered adaptive [26]. While some women are able to manage their fears, an increasing number of women have high levels of fear that may interfere with their mental health and future mother–child relationship [27,28]. 

In this study, we use the term “fear of childbirth” to refer to the severe fear that may be associated to harmful psychological implications for women. While some researchers associate the fear of childbirth with other diseases, such as generalized anxiety disorder or phobias [29,30], other authors consider fear of childbirth as a specific entity [31,32]. 

The literature recognizes the effect of high general anxiety (trait) on fear of childbirth, even emotional fragility, and a vulnerability to depression [23,33,34]. Furthermore, some authors highlighted that fear of childbirth can hide a wider fear of taking on the maternal role: these deep worries are transferred into a more concrete and manageable fear, the fear of childbirth [19,35], which may be associated with stress, anxiety, depression, and lack of social support [31]. 

Research has underlined fear of childbirth to be a strong predisposing factor for post-partum depression, especially in women without a history of depression [36]. Regarding this latter result, we can assume that while women with a history of depression might be more in contact with their emotional difficulties, women with fear of childbirth can express their emotional difficulties through fear of physical pain. 

### The Present Study

This evidence suggests that the pre-partum somatic symptoms and the fear of childbirth can be the first warning signs related to women’s difficulties in acquisition of their own maternal role, that if not picked up, can allow difficulties in the construction of the mother–child relationship during post-partum [37,38,39,40,41,42,43]. 

**Figure 1:**
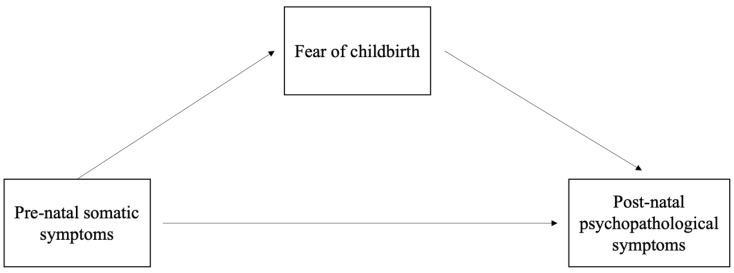
Path diagram of the hypothesized mediation model.

Based on the previous theoretical premises, the present study aimed to extend prior research by assessing the complex interplay between women’s somatic symptoms (such as nausea, vomiting, and daily sleep duration) during pregnancy, fear of childbirth, and post-partum depressive and anxious symptoms. Specifically, while previous studies have investigated nausea and vomiting symptoms jointly, considering them as a single symptomatologic manifestation, no published study has focused on the presence of pre-partum somatic symptoms (separately, nausea, vomiting and daily sleep duration), fear of childbirth, and post-partum anxiety and depression. Furthermore, we considered somatic symptoms and fear of childbirth as two symptomatologic forms linked to the body but in different ways: with somatic symptoms such as nausea and vomiting, mothers can express their worries through the body; with fear of childbirth, mothers direct their worries through an emotion (i.e., fear) toward the body. Finally, this study aimed at taking a step forward in a more in-depth understanding of the role of the fear of childbirth, not only in its direct relationship with some psychological symptoms in pregnant women (as suggested in literature), but also as a possible mediator in the relationship between pre-partum somatic symptoms (i.e., nausea, vomiting, and daily sleep duration) and the post-partum depressive and anxious symptoms. Figure 1 shows the hypothesized model.

## 2. Materials and Methods

### 2.1. Participants and Procedure

Thanks to the collaboration with the pediatric services present in public and private consultancies (health centers and hospitals), *N* = 319 mothers of children from 3 to 6 months of age were recruited. Mothers were turning to counseling centers for their children’s pediatric visits.

From the total sample, we excluded mothers that had current or past illegal drug use (*N* = 5), mothers with pharmacological treatment (*N* = 9), with systemic (gastrointestinal, renal, pulmonary, and cardiovascular system abnormalities) or hormonal diseases that can cause nausea and vomiting (*N* = 3), with previous psychiatric disorders (*N* = 43), and with children with fetal congenital malformations (*N* = 1). 

The final sample was composed of *N* = 258 mothers, from 21 to 55 years of age (average age = 32.26; SD = 5.26). A total of 96.20% of women were married/cohabiting and they had an average number of years of relationship with the father of the child of 8.53 years (SD = 5.17; range from 2 to 35). Most mothers reported their highest level of education to be high school (48.80%).

A total of 70.90% of mothers were primiparous and, 87.20% of mothers stated that the pregnancy was desired. A total of 18.20% of mothers stated that this was a high-risk pregnancy. 

Furthermore, *N* = 32 mothers stated that they have had gestational diabetes, *N* = 9 metabolic disorders, *N* = 36 high blood pressure, and *N* = 22 pre-eclampsia. Moreover, *N* = 16 women had a previous high-risk pregnancy, *N* = 65 had previous miscarriages, *N* = 15 had previous voluntary interruption of pregnancy, and *N* = 38 experienced bereavement or trauma in the past year. 

Regarding children, they were 148 females (57.36%) and 110 males (42.64%). 

Mothers filled out questionnaires about anxiety and depression symptoms (described below); in addition, a sociodemographic questionnaire was administered to assess the course of pregnancy and daily sleep duration, nausea, and vomiting in the three trimesters of pregnancy separately.

### 2.2. Measures

Sociodemographic questionnaire: Mothers filled out a sociodemographic questionnaire assessing pregnancy variables: age at partus, body mass index (BMI), education, employment, comorbidities, history of psychiatric disorders, sleeping habits before pregnancy, domestic violence in present or previous relationship, alcohol consumption, smoking before pregnancy and currently, planned pregnancy, and eventual pregnancy complications. Related to somatic symptoms, mothers were asked to report daily hours of sleep, frequency of nausea (average daily hours of nausea), and vomiting episodes (frequency per day), differently for the three trimesters of pregnancy.

State Trait Anxiety Inventory ((STAI) [44]; Italian version [45]) is a validated questionnaire, composed of 40 items grouped in two scales: state anxiety (STAI-S), is where anxiety is conceived as a particular experience, a feeling of insecurity, impotence in the face of perceived damage that can lead to either concern or escape and avoidance; and trait anxiety (STAI-T), consists of the tendency to perceive stressful situations as dangerous and threatening and to respond to the various situations with different intensities. For the purpose of the present study, we considered only the state anxiety scale. According to the Italian manual [45], the three levels of the state anxiety (STAI-S) are assigned according to the following cut-off scores: low level (scores lower than 31); intermediate level (scores comprised between 31 and 46); and high level (scores above 46). In the present study, Cronbach’s alpha was 0.94 for STAI-S.

Edinburgh Postnatal Depression Scale ((EPDS) [46]; Italian version [47]), is a 10-item questionnaire developed to assist in identifying possible symptoms of depression in the post-partum period. Moreover, it has adequate sensitivity and specificity to detect depression symptoms in the antenatal period [48]. In the Italian version [47] the maximum positive predictive value (PPV) using 12/13 points as a threshold (PPV = 90.90%), with a specificity of 98.9% and a sensitivity of 55.60%, was estimated. In the present study, Cronbach’s alpha was 0.87.

Wijma Delivery Expectancy Questionnaire ((WDEQ-B) [49]; Italian version [50]), is a self-report measure, assessing the intensity of emotions linked to the expectations of the delivery. There are two versions, A and B, which can be administered before and after the childbirth experience. This study used the WDEQ-B. In version B, respondents answer the questions remembering how delivery went. In WDEQ-B, the intensity of emotions is connected to the experience of childbirth, so the women respond to the items with specific reference to lived experience. Internal consistency and split-half reliability of the WDEQ-B are ≥ 0.87 [49]. Moreover, Wilma et al. [49] showed the equivalence of the W-DEQ for comparing antepartum and post-partum scores (both in nulliparous and parous women) also confirmed in an Italian study with women from normative and clinical samples [51]. Its minimum total score is 0 and its maximum total score is 165. A greater total item score indicates more intense fear of delivery after childbirth. Fenaroli and Saita [50] determined that scores above 85 indicate high fear of childbirth. In the present study, Cronbach’s alpha was 0.91.

### 2.3. Data Analysis

Preliminary descriptive analyses were carried out (average scores, frequencies, percentages, and reliability of the measures). Then, after verifying normality of distribution and linearity, we conducted Pearson’s correlation analyses to determine significant correlations between study variables and to identify significant sociodemographic covariates. Based on significant correlations that emerged, mediation analyses were conducted to verify whether women’s fear of childbirth mediated the effect of levels of pre-partum somatic symptoms (i.e., nausea, vomiting, and daily sleep duration) on post-partum state anxious and depressive symptoms. Consequently, three separate mediation models were conducted. Indirect effects were evaluated with 95% bias-corrected confidence intervals (CIs) based on 10,000 bootstrap samples. All analyses were performed using IBM SPSS software, version 26.0. Mediation analyses were conducted used Hayes’s PROCESS macro [52] (Model 4).

## 3. Results

### 3.1. Descriptive Statistics

Preliminary descriptive analyses were carried out. Table 1 shows daily frequencies of nausea, vomiting, and sleep during the three trimesters of pregnancy. As it is possible to see, while in the first trimester *N* = 102 women stated to sleep daily more than 8 h, and *N* = 38 women stated to sleep more than 10 h, in the last trimester *N* = 78 women stated to sleep between 3 and 5 h per day.

Regarding both nausea and vomiting, Table 1 shows that in second and third trimester of pregnancy women without nausea and vomiting increased in frequency, although some women showed nausea (*N* = 17) and vomiting (*N* = 7) also in the third trimester.

Furthermore, descriptive analyses were conducted also on WDEQ-B, STAI-S, and EPDS scores. Table 2 shows average scores, standard deviations (SDs), minimum and maximum scores about fear of childbirth and post-partum anxiety and depression.

Findings showed that *N* = 49 had high levels of fear of childbirth, exceeding the WDEQ-B clinical cut-off (scores > 85). Moreover, *N* = 57 women exceeded the EPDS clinical cut-off (scores > 12). Lastly, for the STAI-S, *N* = 66 women had low levels of anxiety, *N* = 114 women had intermediate levels of anxiety, and *N* = 70 women had high levels of anxiety. 

### 3.2. Associations between Somatic Symptoms, Fear of Childbirth and Post-Partum Anxiety and Depression

To verify possible associations between somatic symptoms during pregnancy, fear of childbirth, and post-partum anxiety and depression, Pearson’s correlation analysis was carried out. Table 3 shows correlations found.

As it is possible to see in Table 3, results showed that vomiting was significantly correlated with fear of childbirth (*p* < 0.01), depression (*p* < 0.001), and state anxiety (*p* < 0.001). On the other hand, no associations have been found between both nausea and daily sleep duration and fear of childbirth, anxiety, and depression (all *p* > 0.05). Finally, fear of childbirth correlated with depression (*p* < 0.001), and state anxiety (*p* < 0.001).

### 3.3. Fear of Childbirth Mediated the Relationship between Vomiting during Pregnancy and Post-Partum Anxiety and Depression

**Figure 2:**
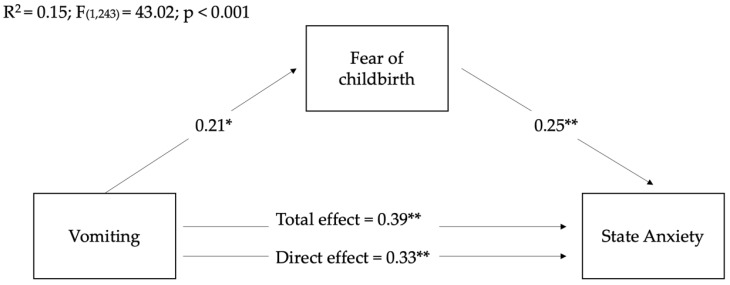
Mediation of fear of childbirth on the relationships between women’s levels of vomiting during pre-partum and state anxious post-partum symptoms. Coefficients shown are standardized path coefficients. c’: direct effect; c: total effect. * *p* < 0.01, ** *p* < 0.001.

**Figure 3:**
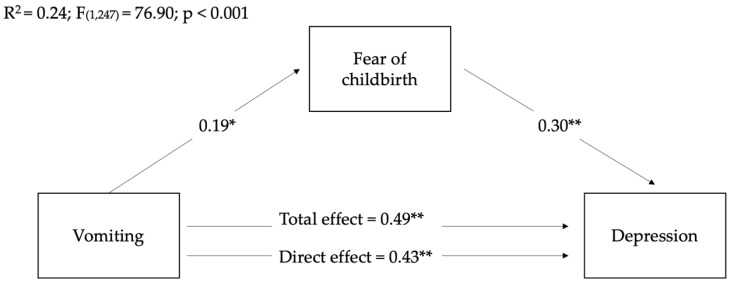
Mediation of fear of childbirth on the relationships between women’s levels of vomiting during pre-partum and depressive post-partum symptoms. Coefficients shown are standardized path coefficients. c’: direct effect; c: total effect. * *p* < 0.01, ** *p* < 0.001.

Based on the fact that, in contrast to our assumptions, no positive correlations were found between nausea and sleep duration and the mediating (fear of childbirth) and outcome variables (anxiety and depression), we conducted mediation analyses to verify whether women’s fear of childbirth mediated the relationships between women’s levels of vomiting during pregnancy on post-partum anxious and depressive symptoms. As it is possible to see in the following figures, the total and direct effects of women’s levels of vomiting on women’s state anxiety (Figure 2) and depression (Figure 3) were significant, showing their partial mediation effects.

Although the three models are all significant, it is possible to see that the mediation model related to the effect of the fear of childbirth on the relationship between vomiting and post-partum depression showed a higher R2 than the other models. This means that the model explained 24% of the variance in mothers’ post-partum depressive symptoms. 

Regarding indirect effects, as it is possible to see in Table 4, fear of childbirth significantly mediated the relationship between women’s levels of vomiting during pregnancy on post-partum depressive symptoms, suggesting its partial mediation role.

## 4. Discussion

The present study aimed to assess the complex interplay between women’s somatic symptoms during pregnancy, fear of childbirth, and post-partum anxious and depressive symptoms. Specifically, we assessed the presence of pre-partum somatic symptoms (separately, nausea, vomiting, and daily sleep duration), fear of childbirth, and post-partum anxious and depressive symptoms, in a sample of mothers with children between 3 and 6 months of age. Descriptive analyses showed that daily sleep duration is higher in the first trimester with respect to the others. Related to nausea, although there was a decreasing trend, in the last trimester almost half of women (48.06%) continued to experience nausea daily; moreover, 16.28% of them continued to have at least one episode of vomiting per day, during the last trimester of pregnancy. These results highlight data slightly higher than those reported in the literature. Indeed, Coronado et al. [53] found that the prevalence of nausea and vomiting in the last trimester of pregnancy was 26.20% and 14.10%, respectively.

Related to psychological variables, findings showed that the 18.99% of mothers reported clinical levels of fear of childbirth. This result is in line with the literature that found a prevalence between 3.70% and 43% of pregnant women were afraid of delivery [54]. Several authors have pointed out that the deep-seated concerns that mothers experience during this time of change are transferred into a more concrete and controllable fear, the fear of childbirth [19,35]. Furthermore, Hu et al. [55] found that fear of childbirth was present in the 34.90% of pregnant women, highlighting high levels of fear of childbirth during COVID-19 pandemic. 

Regarding anxious symptoms, 27.13% of women had high levels of state anxiety, and 22.87% of women had high levels of anxiety. These data are in line with the literature. As a matter of fact, data before the COVID-19 pandemic highlighted that the prevalence of post-partum anxiety disorder was 11.1% [56]; during the COVID-19 lockdown, Suárez-Rico et al. [57] found trait anxiety symptoms in 32–43% of the study participants.

Furthermore, regarding the depressive symptoms, findings showed that *N* = 57 women (22.09%) exceeded the EPDS clinical cut-off (scores > 12). Based on the study by Shorey et al. [58], prevalence of post-partum depression in Europe was 8%, while during the COVID-19 pandemic mothers had more depressive symptoms [59] and clinical scores with the EPDS questionnaire were found in 34–45% of mothers [57].

The objective of the present study was to verify whether the fear of childbirth can mediate the relationship between pre-partum somatic symptoms (i.e., nausea, vomiting, and daily sleep duration) and the post-partum depressive and anxious symptoms. Specifically, we hypothesized that fear of childbirth can explain the relationship between somatic symptoms in pregnancy and post-partum anxiety and depression. 

Results showed that vomiting and fear of childbirth were significantly correlated both to post-partum anxious and depressive symptoms. Daily sleep duration and nausea were not correlated with post-partum psychological symptoms. Regarding daily sleep duration, we hypothesized that this might result from the fact that while some women showed an increase in daily sleepiness, other women reported insomnia, while other women referred lassitude [12]. Our current data do not allow us to understand whether and how differences between sleep-related difficulties experienced by women may be related to increased post-partum psychopathological risk, and further studies are needed to understand this factor in pregnancy. Regarding nausea, to date studies have investigated nausea and vomiting jointly [3,8,9,10], assessing severe or prolonged NVP [14]. In particular, an interesting recent study focusing only on the first trimester of pregnancy found that a combination of high levels of human chorionic gonadotropin hormone (hCG), depressive symptoms, and a history of depression were associated to high levels of nausea and vomiting [60], highlighting the psyche–soma relationship. In the present study we investigated separately nausea and vomiting, and our results highlighted that while vomiting was significantly correlated with fear of childbirth and both post-partum anxiety and depression, nausea was not. In line with Fitzgerald’s results [9], showing that pregnant women who experienced high levels of both nausea and vomiting had more often considered their pregnancy to be unwanted, compared with pregnant women who experienced only the symptom of nausea, we hypothesized that vomiting may represent an expulsive acting out towards the changes that are occurring in the woman’s identity role that, if not understood, may lead to subsequent psychopathological symptoms of anxiety/depression. In fact, the results show that it is the vomiting symptom that is associated with a following psychopathological risk, while there is no such association with nausea, which can be a symptom meant to represent a “normative difficulty”.

Then, mediation analyses showed that fear of childbirth partially mediated the relationships between the levels of vomiting during pregnancy and post-partum state anxiety and depression. This result is very interesting, showing that a physical, bodily symptom (i.e., vomiting) can be an early indicator of a difficulty, which can then lead to fear of childbirth (fear toward/about the body), and later lead to a psychopathological symptom. In line with our previous hypothesis, seeing vomiting as an expulsive acting out towards the women’s changes during pregnancy, we assumed that fear of childbirth can represent a shift toward the body of some women’s worries and fears, as suggested by Fisher, Hauck, and Fenwick [35], and that with vomiting, women directly expel a fear that is not mentalized (and therefore not shifted either). Numerous studies have highlighted the hormonal role on this somatic symptomatology, from a bio-psycho-social perspective, several studies have shown the association between psychological factors and biological mechanisms leading to symptoms such as nausea, vomiting, and sleepiness [4,12,13,17,18,19].

The present study had several limitations. Our research is a retrospective study and, although several studies have considered mothers’ memories of somatic symptoms during pregnancy to be reliable [3,10], further longitudinal studies should confirm our results. Another limitation of the present study concerns the fact that we did not consider other variables that may be protective factors, such as social support [61], emotion regulation [62,63], and paternal mental health [64,65,66], which can be taken as moderating variables in the model we verified.

Despite these limitations, this study had several strengths. First, to our knowledge, this is the first study that assessed the mediation role of women’s fear of childbirth in the relationship between somatic symptoms during pregnancy and post-partum anxiety and depression. Furthermore, this is the first study that assessed nausea and vomiting separately. In fact, most studies to date have considered symptoms of nausea and vomiting as a single symptomatic manifestation, but the results of our study show that it is only high levels of vomiting that are associated with post-partum anxiety and depression. Overall, this study highlights an important link between the symptoms that women express with their bodies during pregnancy and the psychopathological symptoms that emerge in the post-partum period, allowing a reflection of the psyche–soma link in a moment of women’s great identity transformation.

## 5. Conclusions

Our results can have several clinical implications. Numerous studies have underlined the importance to prevent and promptly intervene on post-partum depression [67]. Preventive interventions can help the co-construction of the mother–child bond [68], because the first months of a child’s life are very important for the mother–child relationship development [42]. Our findings highlighted high levels of vomiting to be associated to post-partum anxious and depressive symptoms. Furthermore, fear of childbirth resulted to partially explain this relationship. These results can allow to implement preventive programs for post-partum depression, considering these symptoms as important risk factors.

In order to best implement prevention and/or early intervention programs, we believe that further studies should investigate the findings in greater depth, especially longitudinal studies. 

## Figures and Tables

**Table 1:** Daily frequencies of nausea, vomiting and sleep during pregnancy, divided by trimester.

	I Trimester	II Trimester	III Trimester
**Sleep duration**			
3–5 h per day	21 (8.10%)	33 (12.80%)	78 (30.20%)
6–8 h per day	97 (37.60%)	114 (44.20%)	112 (43.40%)
8–10 h per day	102 (39.50%)	103 (39.90%)	52 (20.20%)
>10 h per day	38 (14.70%)	8 (3.10%)	14 (5.40%)
**Nausea**			
Never	67 (26.0%)	133 (51.60%)	132 (51.20%)
<2 h per day	65 (25.20%)	75 (29.10%)	69 (26.70%)
3–5 h per day	69 (26.70%)	30 (11.60%)	38 (14.70%)
>6 h per day	57 (22.10%)	20 (7.80%)	17 (6.60%)
**Vomiting**			
Never	152 (58.90%)	198 (76.70%)	214 (82.90%)
once per day	59 (22.90%)	40 (15.50%)	32 (12.40%)
2–3 times per day	21 (8.10%)	8 (3.10%)	3 (1.20%)
>3 times per day	24 (9.30%)	10 (3.90%)	7 (2.70%)

**Table 2:** Average scores, standard deviations (SDs), minimum and maximum scores about fear of childbirth and post-partum anxiety and depression.

	Average Scores	SD	Minimum	Maximum
**WDEQ-B**	59.19 ^a^	27.19	13	145
**STAI-S**	40.05 ^b^	12.34	20	80
**EPDS**	8.11 ^c^	5.69	0	27

Note: STAI-S: State Trait Anxiety Inventory, State; EPDS: Edinburgh Postnatal Depression Scale; WDEQ-B: Wijma Delivery Expectancy Questionnaire, Version B. ^a^: average score is lower than the clinical cut-off [50]; ^b^: average score falls in the intermediate level of STAI-S, found by [45]; ^c^: average score is less than the clinical cut-off [47].

**Table 3:** Correlations between somatic symptoms during pregnancy, fear of childbirth, and post-partum anxiety and depression.

	STAI-S	EPDS	WDEQ-B
**Daily sleep duration**	−0.08	−0.12	−0.09
**Nausea**	0.11	0.06	0.03
**Vomiting**	0.39 **	0.49 **	0.17 *
**WDEQ-B**	0.34 **	0.39 **	1

Note: STAI-S: State Trait Anxiety Inventory, State; EPDS: Edinburgh Postnatal Depression Scale; WDEQ-B: Wijma Delivery Expectancy Questionnaire, Version B; * *p* < 0.01; ** *p* < 0.001.

**Table 4:** Indirect effects of women’s levels of vomiting during pre-partum on post-partum depression through fear of childbirth.

Indirect Effect	Effect (BootSE)	LLCI	ULCI
**Vomiting WDEQ-B State Anxiety**	0.05 (0.02)	0.02	0.10
**Vomiting WDEQ-B Depression**	0.06 (0.02)	0.01	0.11

Note: WDEQ-B: Wijma Delivery Expectancy Questionnaire, Version B; BootSE: boot-strapped standard error; LLCI: lower-level confidence interval; ULCI: upper-level confidence interval; all bold values are statistically significant.

## Data Availability

The data presented in this study are openly available in FigShare at https://doi.org/10.6084/m9.figshare.19890106 (accessed on 14 July 2022).
